# A Coincidence

**Published:** 1888-06

**Authors:** 


A COINCIDENCE.

A curious instance of the independent invention of a surgical instrument by two men under widely different circumstances, and for distinctly separate purposes, is revealed by the communication in our April number from Dr. Rollins, and in one from Prof. Busch, Director of the Dental Institute of Berlin, in this. The character and standing of the two men forbid the thought that either should have borrowed his idea from the other, even did the circumstances of the case not do so.

Dr. Rollins is known as an ingenious and original man, who has sometimes been too careless in placing on record in dental journals, where they properly belong, the facts concerning his discoveries, and hence the material which he has delved from the mine of his brain has sometimes been utilized by others, without giving him the credit that is due him. Prof. Busch needs no defense against a charge of unwarrantably seizing the ideas of another. Those who met him at the International Medical Congress last summer, and who listened to his addresses, although delivered in a foreign tongue, were too deeply impressed with his evident candor and honesty, as well as his earnestness and ability, to believe for a moment that he could be professionally dishonorable. Besides, the journal which he sends us proves that he made a record of his invention as long ago as 1884. The facts are undoubtedly just as he states them in his letter to this journal, and it affords another instance of the independent invention of an appliance by two persons.

